# Assisted animal interventions in the ICU: we are responsible for ensuring the well-being and ethical treatment of animals and humans

**DOI:** 10.1186/s44158-024-00200-3

**Published:** 2024-09-09

**Authors:** Maria Grazia Bocci, Daniele Natalini, Rikardo Xhemalaj, Silvia M. Pulitanò

**Affiliations:** 1 National Institute for Infectious Diseases Lazzaro Spallanzani, IRCCS, Rome, Italy; 2 Department of Emergency, Intensive Care Medicine and Anesthesia, Fondazione Policlinico Universitario A. Gemelli IRCCS, Largo Agostino Gemelli 8, Rome, 00168 Italy

To the Editor,

Proof of the effectiveness of animal-assisted interventions (AAIs) in the intensive care unit (ICU) is scarce, but available data show a high potential impact on patient care pathways. Fiore and colleagues emphasized the need for high-quality research evidence, but it is crucial to define ethical, educational, and fields of action [1]. As ethics, safety, and welfare of animals and humans must always be ensured, improvisation is not an option. To this scope, the collaboration between skilled and adequately trained healthcare professionals and other figures, such as veterinarians and animal behavior scientists, will acquire increasing relevance. National guidelines were provided to guarantee safety and standards for AAIs [2]. Many elements can make a difference in improving the quality of AAIs, such as identifying allergies, phobia of animals, or any potential contraindications, and, in the same way, optimizing logistic and organizational aspects [3, 4]. Healthcare professionals involved in AAIs must adhere to strict and specific ethical guidelines. Informed consent should always be obtained, confidentiality must be maintained, and the dignity and autonomy of the individuals must be respected. Human-animal interactions must be respectful, safe, and focused on therapeutic targets rather than a generic consideration of non-maleficence conditions [2].

While planning an educational path, a multidisciplinary approach is mandatory. To provide a holistic understanding of the interventions, professionals with expertise in AAIs, animal behavior scientists, healthcare professionals, and educators need to contribute altogether to the curriculum design and serve as instructors.

It is necessary to establish mechanisms to evaluate the intensive care project’s effectiveness and assess participants’ learning outcomes, including pre- and post-program assessments, feedback surveys, and follow-up evaluations to track participants’ progress and impact.

Finally, collaboration among institutions must be strongly encouraged. Partnerships and collaborations with universities, healthcare facilities, and organizations with proven experience in AAIs allow access to resources, facilitating internships and creating research and knowledge exchange opportunities.

## Data Availability

No datasets were generated or analyzed during the current study.
